# Intimate Partner Violence and Child Custody Evaluation: A Model for Preliminary Clinical Intervention

**DOI:** 10.3389/fpsyg.2018.01471

**Published:** 2018-08-17

**Authors:** Marialuisa Gennari, Giancarlo Tamanza, Sara Molgora

**Affiliations:** Department of Psychology, Università Cattolica del Sacro Cuore, Milan, Italy

**Keywords:** intimate partner violence, separation, divorce, child custody evaluation, relational-intergenerational approach, therapeutic assessment, single case

## Abstract

Intimate partner violence is defined by the World Health Organization as “any behavior within an intimate relationship that causes physical, psychological, or sexual harm to those in the relationship” and it refers to a specific relationship dynamic. In recent decades, an increasing number of studies have focused on this phenomenon, considering its exponential growth over time. Many studies have focused on risk factors for violence within the couple relationship. This paper specifically analyses the association between violence and separation or divorce. Although many interventions have been developed over the years, the effectiveness of extant interventions on violent behaviors is not yet empirically supported. Since clinical experience allows to affirm that both partners can be involved in treatment for intimate partner violence especially during mandated proceedings, the present study focuses on domestic violence in separated couples involved in a child custody evaluation process. In this case, literature supports the need for individualized assessment in order to promote the best intervention according to the specific conditions of each partner, whether the battered one or the perpetrator. However, little research has been done on child custody evaluation in the presence of violent couples. The aim of the present study is to present a model of couple clinical intervention with a separated violent couple in the context of a child custody evaluation. This model can be defined as relational-intergenerational and its main aim is to understand the exchange between familial generations and to search for factors that safeguard and care for family relations. Furthermore, according also to the therapeutic assessment approach, there is an intrinsic connection between assessment and “family transformative potential.” This paper presents the specific working methodology underlying this model, through the description of a single clinical case. In particular, the proposed model provides a multi-dimensional assessment comprising three levels: individual, evaluating parents' history through representations, thoughts, and feelings; interpersonal, investigating the different relations; discussion and dialogue with the parental couple about findings.

## Introduction

Intimate partner violence (IPV) is defined by the World Health Organization as “any behavior within an intimate relationship that causes physical, psychological, or sexual harm to those in the relationship” (World Health Organization, 2012). Specifically, IPV generally refers to a specific relationship dynamic in which affection and aggression are combined (Chester and DeWall, 2018), and violent behaviors occur as an ongoing pattern of abuse (Sugg, 2015). IPV can be non-reciprocal (i.e., perpetrated by only one partner) or reciprocal (i.e., both partners are violent); in the latter case, violent behaviors can occur in different ways (Whitaker et al., 2007).

In recent decades, an increasing number of studies have focused on IPV, considering the exponential growth of this phenomenon over time (World Health Organization, 2013). Research has analyzed outcomes of IPV, focusing on the negative impact of violence on the psychological and physical well-being of partners (e.g., post-traumatic stress disorders, generalized anxiety disorder, depression, health-compromising behaviors, etc.), including over the long-term (Zlotnik et al., 2006; Bosch et al., 2017; Pickover et al., 2017; Spencer et al., 2017). This impact could be mediated by personality characteristics such as temperament traits (Yalch et al., 2017).

The wide dissemination of this phenomenon over the years and the evidence of its negative effects on partners' health underscores the importance of developing interventions for IPV. Specifically, clinicians and researchers are called to develop instruments in order to screen couples at risk for violent behaviors and to prevent the escalation of violence between partners. However, a variety of factors can prevent partners from reporting violence, thus reducing the possibility of access to services and interventions (Spangaro et al., 2016; Gennari et al., 2017).

Although many interventions have been developed over the years, the effectiveness of extant interventions on violent behaviors is not yet empirically supported (Stover et al., 2009). In particular, greater efforts have been made to provide services to support victims, whereas less attention has been paid to intervention programs for batterers (Ferrer-Perez and Bosch-Fiol, 2018). Even less attention has been paid to intervention programs for the couple (i.e., both partners together), also considering that the opportunity for couple treatment is controversial precisely due to the relational asymmetry usual present in violent relations (Beach et al., 2004; Holtzworth-Munroe et al., 2005). In this regard, Kelly and Johnson (2008) suggested the need for effective intervention programs tailored to the specific characteristics of partner violence.

Clinical experience allows us to affirm that both partners can be involved in treatment for intimate partner violence especially during mandated proceedings, that is: (a) in mandatory evaluations carried out by social services in cases of multi-problematic families; and (b) in cases of child custody evaluations. In the first case, social services, after having obtained authorization from appropriate judicial authority, launch investigations and evaluations regarding parenting skills after being alerted by different actors in the social context (school, neighborhood, sports groups) in order to protect children from variously problematic family situations. In the second case, the court requests an intervention in order to supervise the conditions of the couple's separation or divorce.

In this paper we focus on this second condition. Specifically, the aim of the present study is to present a model of couple clinical intervention with a separated violent couple in the context of a child custody evaluation through the description of a single clinical case. In this paper, moreover, we make reference to the type of intimate partner violence that involves acts of verbal and physical aggression (injuries caused by blows and/or objects) because it is one of the most common and clearly identifiable forms of violence (World Health Organization, 2012).

## Violence and divorce

Research has focused on risk factors for violence within the couple relationship, and many variables have been analyzed as predictive of violent behaviors (e.g., childhood experiences and history, socio-demographic characteristics, intrapersonal and interpersonal variables, biological factors) (see for example, Capaldi et al., 2012; Dim and Elabor-Idemudia, 2017; Goodnight et al., 2017; Chester and DeWall, 2018). In particular, this paper focuses on the association between violence and separation or divorce. Some studies underscored that violent behaviors could lead to the breakdown of the couple relationship (Davidson and Beck, 2017). At the same time, separation (or divorce) could be considered as a risk factor because it can make possible the emergence as well as the escalation of violence within the couple (Stolzenberg and D'Alessio, 2007; Toews et al., 2008; Ellis et al., 2014). Considering that divorce in itself is a critical and potentially traumatic event (Cigoli and Scabini, 2006; Parmiani et al., 2012), the presence of violent behaviors between partners makes this process more challenging, increasing the risk of maladaptive outcomes for partners (e.g., depressive symptoms, etc.) (Rutter, 2005) and for children who are exposed to a double stress (Bernet et al., 2016): parental separation and violent behaviors. Furthermore, the presence of violence in couples during the separation process could lead to more negative post-divorce outcome both in terms of general agreements between partners and the co-parenting relationship, raising important issues about child custody (Lessard et al., 2014). The exercise of parental roles also depends on the specific type of violent behaviors (Davidson and Beck, 2017; Hardesty et al., 2017).

Regarding interventions for separated or divorced violent couples, literature supports the need for individualized assessment in order to promote the best intervention according to the specific conditions of each partner, whether the battered one or the perpetrator (Beck et al., 2013; Hardesty et al., 2016). However, little research has been done on child custody evaluation in the presence of violent couples (Saunders et al., 2015). More research is needed to respond to some open questions such as whether the type of violence makes a difference and whether and when shared parenting could be practicable for violent couples (Saunders, 2015).

## Violence and child custody evaluation

Child custody evaluation makes it possible to obtain a clinical space within a social-judicial mandate aimed at the parental couple in order to reorganize the family relations after a separation in the best way possible (Gennari et al., 2014). This mandate is defined by the judge to whom the partners have appealed, asking that “justice be rendered” in a situation perceived to be unjust or prejudicial for oneself or for one's children.

It is important to highlight the unique characteristics of child custody evaluations to understand the possibilities of clinical intervention even when situations of intimate partner violence exist. This context is characterized by transfert (from the couple to the judge) that should be correctly understood and considered (Cigoli, 1998). The characteristics of the partners' petition to the judge, contained in the court proceeding documents, always have important meanings concerning the partners' needs and fears as well as their goals and objectives, of which they are not always conscious. In any case, the consultant as the judge's competent and trusted expert, accepts from both the judge and the couple the task of rendering justice, acknowledging and establishing rights and wrongs. This is the particular intervention setting that, differently from what usually happens, makes it possible to eviscerate and treat violence and enables the partners to entrust themselves to the consultancy precisely in the hope that the wrongs they have received can be rectified. We can thus affirm that this specific intervention setting promotes the trust that one needs to be able to expose one's pain and suffering, including that of violence. It must also be added that in these situations, the judge has preliminarily directed that the partners live separately, often imposing a certain physical distance (restraining order) between them: this is an element that gives the partners the necessary peace of mind to be able to work with the clinic, reducing the fear of violent reprisals.

The purpose of the child custody evaluation is to provide the judge with useful information for establishing the best living conditions for the children as he/she decides on custody, residence, and visitation rights between children and the parent with whom they do not live on a daily basis. Thus, this is a parenting assessment intervention. In this context, the partners often ask to have custody of the children, and for this very reason they are highly motivated to convince the consultant of their good behavior, both as a person and a parent. In this scenario, therefore, it is not uncommon for the parents to disclose incidents of violence with particular vehemence and in great detail, even when these actions did not occur. In short, we can affirm that the child custody evaluation is an assessment setting where violence is brought up very naturally and is often accentuated even as a means to getting custody of the children. In cases of violence, it is thus important for the consultant, even before evaluating the resources and problematic aspects of the partners and their relationship, to evaluate three aspects connected to violent behavior: power, model, and primary perpetrator of the violence (Jaffe et al., 2008). Distinguishing between various types of violence makes it possible to evaluate its seriousness and thus the risk for the children as well as the necessity of putting into place protective mechanisms for the child in the custody decision process, and at the same time to understand the couple dynamics of violent behaviors.

In this regard, for example, some authors (Lebow and Rekart, 2007; Jaffe et al., 2008; Kelly and Johnson, 2008) identified four types of violence in the context of child custody evaluation: (1) Abusive-controlling violence (ACV), also called battering or intimate terrorism or coercive controlling violence, that is, the use of coercive behaviors (e.g., threat, force, emotional abuse, etc.) by a partner to dominate the other inducing fear, submission, and compliance; (2) Conflict-instigated violence (CIV), also called situational or common couple violence, that is, the perpetration of violence by both partners who have limited skills in resolving conflict; (3) Violent Resistance (VR), that occurs when one partner uses violence to defend in response to abuse by the other partner (it may be a self-defense reaction or an overreaction); (4) Separation-Instigated Violence (SIV), that is, when either a man or a woman perpetrates violent behaviors as a reaction to the stress due to divorce in a relationship that has not otherwise been characterized by violence.

It is clear that in the first case (ACV)—in which men are usually the offenders and women are the victims (Kelly and Johnson, 2008)—it will be very difficult to conduct a child custody evaluation that can function as a preliminary clinical intervention able to treat intimate partner violence; in the other cases, instead, the child custody evaluation can be considered to be efficacious as a first intervention to assess and treat the partners' violent behavior.

The longstanding clinical experience of the authors of this contribution confirms the possibility of working with partners conjointly in the (2), (3), and (4) situations of violence as defined by Kelly and Johnson. These are situations in which the violence has the following characteristics:

1. It is a temporary behavior and is specifically connected to the separation event; thus, it has not always characterized the couple relation. Or;
2. It is a behavior undertaken by both partners, even if in different quantities or forms, and thus a certain reciprocal tolerance/use of violence is found in both partners. This entails the presence of a certain equilibrium and shared contribution on the part of the partners in reciprocally constructing their violent relation, as well as a distribution of responsibility with respect to the violent behavior. These are cases in which both partners are, at least in part, both victims and perpetrators;
3. It is a behavior that does not assume the most extreme forms of violence, at least in the partners' intentions, or else the intention of eliminating the other partner never arises.

As we shall see in the clinical case presented herein, the evaluation of the situation of violence takes place in the first assessment level with respect to the tolerance and exposure of each partner to violence. In the second assessment level, which has to do with the couple relationship, the forms of violence and the reciprocity of violent behaviors are investigated in order to verify the shared responsibility for the violence. In cases in which, from the first joint meeting with the couple, the clinician finds responsibility for the violent behavior in one partner only and the total victimization of the other, or in cases in which the impossibility of a reciprocal dialogic-interactive exchange between the partners is apparent in the first joint child custody evaluation encounter, the assessment levels will be carried out in individual, and not joint, encounters.

It must be pointed out that while there is agreement in literature on the need for an initial differential diagnosis of the type of violence occurring, to date, there do not appear to be specific instruments for such an evaluation in the child custody evaluation setting. Moreover, although many IPV screening tools have been developed over the years (Crane et al., 2017), most were evaluated only in a small number of studies (Rabin et al., 2009), so that it is up to the clinician's theoretical and methodological competency to evaluate the severity and dynamic of the couple's violent behavior.

## Proposal of a model for work with violent couples in child custody evaluations

In what way can a process of parenting assessment be considered a possible preliminary clinical intervention with the couple, in situations with IPV? To achieve this objective, we believe it is indispensable to develop a specific clinical work methodology defined as the relational-intergenerational approach to child custody evaluation (Cigoli and Scabini, 2006; Gennari et al., 2014; Ranieri et al., 2016). Its main characteristic is that it is aimed at understanding the exchange between familial generations and at searching for factors that safeguard and care for family relations. The consultant is thus called upon to perform actions that considers multiple aspects for the specific purpose of offering the judge the most complete report possible regarding the parents, their children, and their relations. A consulting framework is therefore necessary, one that traces the subjects' history, finds the meaning of events, and captures the characteristics of people and their relations so as to open up the possibility of a future organization that can safeguard the minor's development.

In this model of child custody evaluation there is an intrinsic connection between assessment and “family transformative potential,” a connection that is also at the basis of a therapeutic assessment approach (Finn, 2007) and that, therefore, goes well beyond the production of a static snapshot of the participating subjects (Gennari and Tamanza, 2017). In fact, knowledge of the people and their relations is connected to the dynamic of the separation, as a transformation occurring in the way family is lived during the separation. Thus, it is not only a matter of capturing the marital and parenting dynamic, but also of assessing the capacity of the family configuration to evolve as it copes with the separation transition. Thus, the consultant cannot do without some sort of prognostic apparatus that is founded on a temporal perspective connecting past, present, and future.

Interest in the dynamic and process aspects requires a specific working methodology which, by utilizing specific skills and instruments, activates and moves the family so that, in addition to having evaluative information, it is possible to consider and activate the potential to transform and, thus, also care for the family relations. In short: without eliciting a change, to any degree and in any direction, it is impossible for the consultant to predict the family's possible evolution and to suggest to the judge what might be the family's potential in regard to the development of new forms of family relations to be defined with the separation.

The transformative potential in the relation is solicited from the parents' ongoing participation and reflection on the information coming to light during the clinical process, as well as from the possibility of ultimately agreeing with the parents on solutions to be presented to the judge, precisely due to the growing awareness of the parents themselves during the entire child custody evaluation process. It should be clarified that any soliciting of possible solutions to the parental conflict (included how to manage the violent behaviors) is, first of all, a diagnostic operation and not an obligation to transact or negotiate. In any case, it represents an opportunity: for the consultant to discern additional characteristics in the parents, while for the parents it is an opportunity to take into their own hands the parental function in its aspects of decision making and planning. From this perspective, the partners' resources are activated and valorized from the start, and directed toward behaviors and actions of constructive change.

Within this work model, assessment consists of a multi-dimensional investigation comprised of three different levels:

1. the production of information on an individual level through an evaluation of the parents' representations, thoughts, and feelings;
2. the production of information on an interpersonal level through an investigation of the different relations: that between the partners, of each parent with the children, of the parental couple with the children, and of the entire family system. To manage this level of assessment, the Clinical Generational Interview (CGI; Cigoli and Tamanza, 2008) was used to explore the couple relationship. It is a semi-structured interview during which the two partners are asked together to describe their relationship as a couple. In particular, this interview—which is structured around three different thematic axes (family of origin, couple relationship, and parental relationship)—allows one to better understand the quality and characteristics of the family relations through 21 open-ended questions and 2 sets of paintings. This instrument included a double coding system that makes it possible to classify families with respect to generativity, distinguishing between positive, negative, and critical situations. The principle focus of CGI is: the meanings of the partners' choices, the aims of the couple relationship, the outcome of the relation in regard to meeting each partner's needs and desires, the impact on the couple of disillusion regarding the unsuccessful relational outcomes, and each partner's coping with the couple's relational failure;
3. discussion and dialogue with the parental couple about findings. Specifically, this assessment level is aimed at acquiring an understanding of parenting not only as a quality of individual intrapsychic functioning or the parents' personality characteristics, but from a systemic perspective of interrelation between the two parents, the child, and the relational systems of the family of origin. From this perspective, the evaluative criterion of parental adequacy will not be limited to considering the “care-giving capacities” of each parent, but will center attention, in an environment of reciprocal relation, on each parent's capacity and willingness to realize, maintain, and consolidate “parental unity” and, more broadly, family unity in order to safeguard the minor's family (New York Convention, 1989)^1^. With these specific characteristics, the child custody evaluation context makes it possible to offer an assessment of the parents and also of the violence in the parental couple while constituting a preliminary treatment phase of the parental conflict, including the aspect of violence that characterizes the relation.

In line with the specific aims of the present work, in the following paragraphs we will exclusively examine in depth the assessment of each partner and of the couple relation in order to explore treatability with respect to violence. Thus, we do not discuss all the assessment levels typical of the child custody evaluation.

## Evaluating and treating couple violence through the child custody evaluation: the case of james and mary

Each partner signed the written informed consent form given by the clinician at the beginning of the child custody evaluation process for the processing of data for research and scientific dissemination purposes. The written informed consent form is prepared in accordance with the national law on the processing of personal sensitive information and privacy, pursuant to Article 10 of Law no. 675/96 and subsequent modifications. Since this is not a research project, ethics approval by an institution was not required as per university guidelines. Nevertheless, to preserve the confidentiality of the reported clinical case, the authors did not describe details which would make the couple recognizable.

### The first assessment level: individual characteristics of each partner

Literature underscores the importance of intrapersonal variables in the study and understanding of violent behavior. In particular, perpetrators of IPV often present some symptoms of psychopathology such as antisocial or borderline personality disorders (Chester and DeWall, 2018). Some psychological traits could also be considered as predictive factors/risk factors for IPV (Ulloa et al., 2016; Goodnight et al., 2017). For example, a lack or a fatigue in self-control and self-regulation is an important predictor of IPV perpetration (Finkel et al., 2009; Chester and DeWall, 2018). Furthermore, other personality characteristics (e.g., narcissism, neuroticism, etc.) were described as predictive of violence, both for perpetration and victimization although with some gender differences (Larson et al., 2015; Talbot et al., 2015; Ulloa et al., 2016). Finally, couple violence is connected to mood states. In particular, emotional instability could predict IPV (Talbot et al., 2015), and, at the same time, violence improves a deep emotional instability in the people involved (Beach et al., 2004; Pickover et al., 2017).

Considering that IPV is a social process, is very important to assess the partners' interpersonal adjustment (i.e., skills, attitudes, and behaviors that an individual uses when entering a relationship with others). Indeed, lacks and difficulties in interpersonal adjustment between partners (e.g., de-humanization, infidelity, rejection, etc.) could be associated with a violent relationship (Chester and DeWall, 2018).

The history of the individual partners' families of origin is also important for understanding violence in the couple relation. Literature highlights that there is an intergenerational transmission of violence—the so-called “cycle of violence” (Widom, 2017). Specifically, childhood victimization and abuse or childhood exposure to domestic violence are predictors of IPV during adulthood both for perpetrators and victims (Ehrensaft et al., 2003; Dim and Elabor-Idemudia, 2017; Lieberz et al., 2017). In this connection, it thus becomes important to specifically capture the partners' representations of their history, the characteristics of their internalized parental models, and, more in general, their representations of the upbringing context as well as the internalized characteristics of the parental relations experienced by the individual partners as children (Delsol and Margolin, 2004; Skuja and Halford, 2004).

*As shown in Supplementary Figure 1 and Supplementary Table 1*, *James' individual assessment reveals a personality (Questionario di Adattamento Interpersonale—QAI; Di Nuovo*, *1998*) *characterized by pathological impulsivity and narcissism and a problematic mood profile (Profile of Mood States—POMS; McNair et al.*, *1971**; Farnè et al.*, *1991**) with respect to five of the six states investigated (fatigue, tension, depression, vigor, and anger) while the sixth (confusion) is at the limit. It is interesting to observe the scores under the range of normality relative to anxiety and stress in social situations. Undoubtedly, the conflictual separation that James is experiencing influences his moods, yet these aspects have characterized James for a long time, as the story of his adolescence, as well as his marital history, reveal. James narrates that his childhood is characterized by economic well-being and considerable physical comfort; nevertheless, he talks about a constant lack of affection and care in family relations. From James' story, in fact, an almost total absence of significant relationships and relations between each family member emerges: both between the parents, who often betrayed each other even in the presence of the children, as well as between the siblings, who grew up without interactions and significant relationships. In the family context that James experienced, the needs of the family's components did not have space either to be expressed or addressed, except for the sake of appearances. This affective deficiency came to be structured in James in terms of unresolved needs and great anger and rancor toward his family of origin, still today at the basis of rancorous interactions with his family and a feeling of being entitled to compensation for what he feels he missed. The feeling of affective emptiness is well-expressed by high levels of narcissism as well as of impulsivity, which appears to characterize James' relational modalities in his relations with his peers, in his family, and in the couple relation. Moreover, a concern for the social dimension and appearances turns out to be excessive, notwithstanding that these very aspects were abundantly criticized by James in his narrative about his family of origin. His internalized models of marital relations show his father to be domineering, with no significant relation with his own family of origin nor with his wife's family of origin. The mother is described as a woman interested only in her own well-being and disinclined to establish sincere and honest relations with others. The models of marital relations experienced by James are characterized by the possibility of buying affection with money, by the right to use what is given against the other person to keep the other person him/her tied to oneself, in order to meet one's needs, even by force. James' aggressive and violent behaviors can thus be traced throughout his relational history*.

*In Mary's story of her childhood and adolescence, affective relational dimensions are present even if, as she herself says, there was aspects of painful emotional nature. One example is the experience of anorexia in adolescence and young adulthood and an obsessive-compulsive disorder that caused her to get out of bed repeatedly during the night to take a shower, consequences of repeated intrafamilial episodes of violence. Mary talks about a life experience characterized by a male chauvinist mentality in which the female figure is considered as an object to be relegated exclusively to the domestic domain, with no right to express her own needs and desires. Mary's story reveals an experience of precocious adultization (she had to take responsibility for managing the home, her father, and her older brother during her mother's prolonged absences caused by depression and joint problems of the back) and a context particularly lacking in emotional care, which led to the development of an unstable mood (still present in constructs of tension, fatigue, and confusion) and a high score for depression and lack of strength and vigor (POMS). From the point of view of personality traits, there is a diffuse difficulty in coping connected to a lack of assertiveness, that is, to inadequacy experienced in responding to the events and requirements of the context. Her indices for stress and anxiety in social situations are below the threshold of the normal range (QAI), indicating a inhibited and defensively intractable aspect. During the interviews with the consultant, Mary also struggles to stay in dialogue: she is more preoccupied with finishing what she has to say than establishing a dialogic exchange in keeping with the questions posed to her. The parental model experienced is characterized by an abuse of power on the part of the father with respect to the mother (who is forced by her husband to stop working and to meet all of his demands), extensive use of corporal punishment with the children when parental rules were transgressed, and, finally, the parents' legitimization of the brother's violent behavior toward Mary, the younger sibling and a female. Regarding her parents' life together as a couple, Mary says that she would not have wanted to have a married live like theirs, because she would not have been able to bear being an objectified, enslaved, and used woman like her mother. The family relations were characterized by the absence of relationships with the paternal relatives, while with the mother's family there were more interactions and contacts*.

### The second assessment level: the nature and characteristics of the couple relation

The second level of assessment aims to identify the characteristics and quality of a violent couple relationship, beyond the partners' individual differences. The theoretical model presented above assumes that the individual perspective is not sufficient for explaining the couple relationship and, in particular, violent behaviors within the couple. It considers the couple relationship as something unique and specific, as a third part with its own characteristics that must be investigated with specific instruments (Cigoli and Scabini, 2006; Cigoli et al., 2014). The couple relationship originates and is built upon the histories of individuals, according to each partner's needs, desires, and aims regarding the relation. Thus, the couple relationship assessment attempts to capture the specific modalities of establishing and being a couple, underlying those dynamics that are shared and involve both partners as they contribute to the construction of the same relation (Cigoli et al., 2014).

Literature underscores the importance of capturing the specific violence dynamic within the couple (Chester and DeWall, 2018).

*The relation between James and Mary turns out to have been conflictual from the start; it originated as an opportunity to experience a different way of life from the one the partners had lived in their respective families of origin. Mary hoped to feel liberated and more valued as a person. James chose Mary because she was a simple girl, a “housewife,” appreciating her meekness and sweet nature which compensated for the internalized representation of his mother as distant and emotionally unexpressive. The bond, as described by the ex-spouses, is continued until it takes on the characteristic of reciprocal dependency and fusion in which each partner devoted him/herself to meeting the other's needs and healing the pain each experienced in the family of origin. We thus can recognize a relation that originated with the aim of medicating, compensating, and making up for the deficiencies experienced in the partners' history as children*.

*The relational dynamic between the partners, characterized by control and dependency, gave rise over time to a progressive escalation of conflict and aggression that was accentuated by and eventually exploded with the arrival of children. Parental responsibilities, in fact, limited the time and possibility of attending to and indulging reciprocal needs and requests, and the promise of attention began to go unfulfilled. The fact that each partner had limited resources for analyzing the new family configuration in a realistic way, by scaling back personal requests, for example, brought this couple to a condition of stalemate and profound crisis. Each partner had become disillusioned in their initial expectations, and a feeling of real betrayal characterized the experiences of both spouses. Mary felt that she was not appreciated and sought out by her husband, no longer the center of his attention; James says that his wife's unhealthy and obsessive control had increased, and arguments characterized by violent verbal and physical attacks by both partners had intensified*.

*Mary decides to separate from her husband after the discovery that he had a stable extramarital relationship; nevertheless, the couple continues to live together for 3 years without reaching an agreement on a separation. In this period, the reciprocal violence, both physical and verbal, further increases despite the partners' repeated attempts to restore the peace. Both highlight the strength of the bond that unites them still today, the impossibility of accepting the end of the relation, and assert that they cannot tolerate that their partner could have a new relationship and that the children would have to live with someone else*.

*The feasibility of separation for this couple appears to be an impossible goal that is greatly complicated by each partner's unconscious actions to prevent the other one from leaving. The outcome is an even more unstable and violent relational situation. It is more than evident, still today, that the spouses are not able to separate despite the violent behavior and the reciprocal harm inflicted in 14 years of their relationship as a couple. In brief: James and Mary are living the impossibility to accept to lose the Other (spouse and children) because he/she has the function to care the one's own aspects of pain. For this reason partners are moved to force and obligate the Other, also through violent behavior, to answer one's own vulnerabilities and frailties*.

### The third assessment level: the search for resources for abandoning violent relational modalities and achieving the marital separation

The information gathered with the prior assessment levels allows us to capture the specific meanings of the violence in James' and Mary's story.

Since childhood, both experience violence as a relational modality. Many aspects of their couple relation is also characterized by demands for salvific support which, not having been fully reciprocated since children arrival, fostered aggressive, and violent behaviors. Nevertheless, the partners are unaware of these aspects, even if they are present in their narratives; that is, they are not able to grasp to what extent some of their personal characteristics and relational modalities are dysfunctional and are thus the cause of reciprocal suffering and the failure of the relation. Each partner, in fact, is only focused on his or her own pain, on his or her own needs. The capacity for grasping the other's struggles is non-existent; the possibility of evaluating how much their own requests are unrealizable and idealistic is completely missing. The representation of the other appears limited to an instrumental function with regard to one's own needs. The literature clearly highlights the risk of exclusively ego-syntonic relational modalities, both for relational failure as well as for situations of violence.

It is therefore the consultant's responsibility, in our view, to “be able to read” and to “help to read” the violence and failure of the relationship in their less conscious meanings as impossible and idealized requests, in addition to promoting a perspective on reading events that also contemplates the other, assigning value to him/her, considering his/her needs and fragilities. Sharing with each partner what emerges from the preliminary assessment levels is therefore the premise for being able to treat the violence. It is a matter of enhancing each partner's reflective functioning and mentalization capacity, which, we know, are often reduced in violent situations (Stover and Coates, 2016). In situations of violence found in separations, the process of elaboration is indispensable for a true emotional separation. Separation, in fact, allows the partners to grow because it introduces them to the process of individuation, allows them to experience possible modalities of independence from the other one, and leads to a complete psychological separation. In reciprocally violent couples, the difficulty of the process is even clearer because the primary processes of individuation and separation have never been accomplished (Gray, 2004).

The clinical work of assessment does not happen because of a spontaneous request on the part of the couple: their willingness, therefore, to reflect, rethink, and put into discussion their relational modalities and behaviors is quite limited. Nevertheless, there are some aspects that make this work possible. The first is precisely the thirst for justice with which each partner arrives in the consultant's setting: being able to demonstrate that one is right and finally finding someone to acknowledge one's claims with respect to the partner. These purposes predispose the spouses to narrate their personal stories and make their own case. This aspect, together with the need for a space where they could feel accepted and express their pain, made it possible in their case to open a space for elaboration.

*The work with James involves reflection on his difficulty with “being alone” and the deep and longstanding conflict in his relations with his family of origin. All of this does not allow him to find his own stability. His family history, moreover, has determined the absence of an affective reference point that has prevented him from perceiving support: the relations that did not “nourish” from an emotional standpoint did not allow him to construct and make use of a relational model of reciprocal exchange: from this also derive the aspects of dependence (which manifest in the counter-dependent modality) that lead him to establish relations in which he demands too much from his partner (but also from others) to be able to satisfy and fulfill his own affective needs. In the profile of the affectively dependent personality, one also finds mood swings that sometimes lead him to lose control of his own reactions toward people who oppose him. Work on the deep anger that he feels when encountering a relational frustration is revealed to be necessary if he is to manage relations with others more calmly*.

*The work with Mary addresses two aspects: her affective dependence and the difficulty of managing her anxiety and sense of incompetence and helplessness. Having grown up alone, without any protection or guidance, exposure to violence and a precocious adultization determined the need to constantly lean on someone in order to feel whole and worthy; indeed, it is precisely this need that does not allow Mary to end her couple relation and, in general, induces her to stay strongly anchored to relations, even dysfunctional ones. The second aspect, closely connected to the first, is the deep social and psychological aloneness in which she finds herself. This dimension of isolation, in fact, leads her to accentuate even more her physical, economic, and relational dependency*.

Working through what emerged from the preliminary assessment phases proceeds through a necessary, if difficult, rereading of the couple relation that allows the partners to become aware of the needs and desires that were not met in the marital relation and the events that made it impracticable to move beyond them. In fact, if the pact of trust underpinning the partners' choice enters into crisis, and the affective theme that had given rise to the bond is not adequately addressed and worked through by the couple, an emotional blockage occurs. This blockage pervades the couple relation, amplifying, and exasperating aspects of conflict and violence and making the process of separation more difficult. Indeed, everything that remains unacknowledged regarding the relation's characteristics will inevitably be transferred and projected on to the parenting dimension, with the consequent and unavoidable involvement of the children. Only by working through the end of the couple bond, therefore, can the parenting dimension be relaunched (Gennari et al., 2014).

The work on James' and Mary's relation was made difficult by the fact that the partners' experience of mourning and loss has particular characteristics: in fact, it is a bereavement where the object is alive and often very present in the real lives of the partners (Losso, 2003). This amplifies conflictual feelings and affections activated by the separation event. Very briefly, the process of critical reflection on the part of the two spouses/parents, to be functionally useful to the process of elaborating the relation, must necessarily entail an assessment of the experience of the relation by means of two psychological actions. On the one hand, the partners are called upon to assume specific responsibilities. This entails feeling part of the history and events that led to the end of the relation that resulted from their own principal modalities of living, acting, and investing in the relation. The goal is to abandon a vision fixated on one's own pain and the lived experience of having suffered an injustice in order to reach a position of shared fragility and responsibility. On the other hand, one must be able to acknowledge the good received from the other and the relation (Cigoli and Scabini, 2006). In this way, in one's relational experience it becomes possible to acknowledge not only the other partner's debts toward oneself but also the credits as a way of offsetting losses and suffering. Thus, the separation relation can find closure with the perception of co-responsibility, and not only of failure.

This is primarily an ethical matter that has to do with the feeling of injustice invoked by both partners at the beginning of the child custody evaluation and makes it possible to put into perspective the experience of being wronged and one's demands for reparation from the other partner. If this does not happen, an unnecessarily persecutory, fragmentary, damaging, and disintegrative approach will prevail, and the conditions for an attack on the family's relations overall, and not only on the martial bond, will take shape. James' and Mary's story exemplifies this aspect: the escalation of violence is the outcome of the non-comprehension of the relation's failure; it is the cipher of the impossibility of accepting that the other has not been able to satisfy one's needs and requests since the beginning of the relation. In fact, themes of loss and quite significant narcissistic wounds emerge preventing the separation (Losso, 2003).

*In the clinical work with James and Mary present together, the aim is to share the affective dependency that characterized both spouses, although in different ways. It also entails eliciting from each partner a request for support from the other, asking him/her for total dedication and care for the wounds experienced as children. Moreover, the possibility of putting into perspective the idealized representation that each one has constructed of the other's role is explored: with James, this involves putting into perspective his expectations of Mary as wife and mother so that he can revisit and possibly recuperate the relationship with his own mother, but also so that he can adopt a position of greater acceptance of Mary's characteristics and modalities. With Mary it is important to put into perspective the salvific omnipotence constantly required of James as husband and father*.

An additional aspect that allows the couple to work in terms of reflection and personal development is each partner's desire to be able to protect and care for the children. It is precisely for the good of the children that the parents are able to move beyond their marital strife and become involved and motivated in the work of discussion and change with respect to themselves. The child is often of such immense value that it becomes possible to discuss even very solid and rigid relational positions and modalities: the child enables the parents to tune into an object other than their own pain; the child's struggles and needs are more legitimate than those of the other spouse and sometimes even than one's own. In fact, the child is the possibility of an opening onto a different future, one that is more positive, and this often motivates the parents to make efforts and pursue goals that are unthinkable if they are focused only on themselves. In short, the child can be an important engine for personal change. But children, as Lemaire (2002) emphasizes, constitute a living testimony of the other parent's presence and make it impossible to erase all traces of the relation, as the partners wish they could do. The children and their needs are thus the starting point that makes it possible to revisit one's representation of one's partner and his/her value as a parent (Cigoli and Scabini, 2006).

*In this regard, discussion with James addresses his operative competence in the relation with his children, his capacity for containment and control, but also the absence of a more affective and supportive parental component. This explains the children's fear of him and their jealousy toward his companion, whom they see as receiving all their father's attention and affection. Moreover, James's difficulties tolerating and, as a result, valorizing at least partially his children's mother are discussed. This aspect also becomes crucial for interrupting the relational dynamic that characterizes the oldest child who, in order to protect his mother, has rigidified into a position of rejection and defiance toward his father*.

*Work with Mary focuses for a long time on how her unstable emotivity involves her children who participate excessively and directly in her malaise and fragility. Her dysfunctional involvement in the conflicts of her husband's family is also problematic since it foments the conflict with her children's father. As a parent, she needs to focus on relational and child-rearing modalities with her children that are less confusing and more evenhanded. Thus, space is made for the children to find in their mother an autonomous and solid parental figure, able to guide and protect them, interrupting Mary's current dynamic in which she is experienced and perceived by her children as a peer in need of protection*.

It is precisely in the presence of both parents that it becomes possible, in a setting of cooperative work, to delineate the children's needs, needs that in the first place point to the importance of the joint exercise of parental functions (Emery, 2012). Indeed, some studies have emphasized that the negative effects of divorce as well as of violent behavior between partners on children's adjustment could be mediated by stable and cooperative co-parenting, which reduced the perceived parental distress (Molgora et al., 2014; Bernet et al., 2016).

## Conclusion

The aim of the present contribution was to present a clinical model of intervention with divorced couples experiencing IPV in the context of the child custody evaluation. Professionals often use traditional work modalities with no adjustment made for the presence of violence (Saunders, 2015). We believe, instead, that in these situations it is important to design a specific intervention that makes it possible, first of all, to evaluate what type of violence is present in the couple (Kelly and Johnson, 2008) and then to work not only in order to eliminate the violent behavior, but also to transform family relations in a more radical way.

In this sense, the model we have described, within a legal framework, could be described as a clinical intervention because it focuses not only on the evaluation of each partner (“the best parent”), but above all on the relationship between the partners. Indeed, bonds cannot be dissolved and erased, but only transformed (Cigoli and Scabini, 2006) and so, even if the partners are no longer a married couple, they will be a parental couple forever. The goal of this intervention is to make each parent an active protagonist able to collaborate, support, trust, and legitimize each other in their parental role. This process of acknowledgment and legitimization of the other is possible only by working to strengthen capacities for mentalization and reflective functioning (Aschieri et al., 2016; Stover and Coates, 2016). This increase the possibility that the partners will acknowledge their own part of the responsibility for the conflict and the violence, and thus initiate actions aimed at a constructive management of the conflict itself, finding the resources needed to care for the children. We know, in fact, that divorce, especially in its more conflictual and violent forms, often risks creating a family scenario in which suffering, reciprocal annihilation, and demands made between the spouses saturate every space, leaving in the background the children's developmental needs and requirements.

In this scenario, a crucial role is played by the dimension of time. In the clinical work, in fact, the consultant, in addition to considering the present, also focuses on the past (historical dimension) and the future. In particular, the future does not only have to do with possible family configurations after the divorce but also with the developmental trajectories of the children involved in the separation. Identifying spaces for change in the parent-child relations and the adoption of future scenarios centered on the children's needs and requirements becomes a primary objective in this intervention model.

This is what happened in the story of James and Mary where both, precisely due to the work of assessment described above, were able to move beyond reciprocally vindictive relational modalities centered on their own childhood needs to collaborative and more reflective modalities oriented principally toward responding to their children's needs. Such a problematic situation required that both partners move away from their reciprocal aggressive and violent relation and concentrate together on the need to help and support their children. From this perspective, it became possible for James to spontaneously undertake a path of personal psychotherapy, and, at the end of the child custody evaluation, both parents agreed to be involved in a joint program of parenting support. This outcome well represents the act of justice that both partners must institute and pursue to give a new reason for hope and redemption to their family relations.

Despite the innovative focus of this method, it presents some limitation. In particular, the work method presented reveals itself to be useful in those specific situations of violence that erupt from the post-separation event and which, therefore, are not prevalent in the couple relationship. The method also showed a certain efficacy in situations of reciprocal violence, even if in different forms and intensities between the partners, as long as these are not extreme situations that put the partners' lives at risk. This assessment proposal has not been sufficiently tested in cases of extreme forms of violence or in situations in which one partner is clearly dominated and victimized by the other. Therefore, in these situations, the proposed assessment method cannot be considered reliable and effective.

## Ethics statement

Ethics approval for this research was not required as per the Università Cattolica del Sacro Cuore's guidelines and Italian laws and regulations. Written informed consent was obtained from all research participants and form the parents/legal guardians of all children. The written informed consent form is prepared according to the national law on the processing of personal sensitive data and privacy, pursuant to Article 10 of Law no. 675/96 and subsequent modifications. This was given to the parents by the clinician at the beginning of the child custody evaluation process, for the collection and processing of data for research and scientific dissemination purposes.

## Author contributions

MG contributed to the writing of the manuscript's clinical case paragraphs. GT supervised the entire manuscript. SM contributed to the writing of the manuscript's first five paragraphs. All authors reviewed and approved manuscript for publication.

### Conflict of interest statement

The authors declare that the research was conducted in the absence of any commercial or financial relationships that could be construed as a potential conflict of interest.

## Footnotes

^1^https://www.ohchr.org/en/professionalinterest/pages/crc.aspx
