# Supplementary material for: Intimate Partner Violence and Child Custody Evaluation: A Model for Preliminary Clinical Intervention
Source: Front Psychol. 2018 Aug 17;9:1471. doi: 10.3389/fpsyg.2018.01471 (PMC6107842; doi:10.3389/fpsyg.2018.01471)
Supplement: Supplementary file 1 [file Table_1.DOCX]

Table 1. Mary and James mood state (POMS)

|  | Mary | James | *Normal Range* |
| --- | --- | --- | --- |
| FATIGUE | 16 | 18 | *(2-10)* |
| TENSION | 25 | 23 | *(4-15)* |
| DEPRESSION | 22 | 20 | *(0-17)* |
| VIGOR | 2 | 4 | *(10-21M; 9-20F)* |
| CONFUSION | 15 | 12 | *(4-12)* |
| ANGER | 18 | 35 | *(8-22)* |
