# Supplementary figures and images for: Intimate Partner Violence and Child Custody Evaluation: A Model for Preliminary Clinical Intervention
Source: Front Psychol. 2018 Aug 17;9:1471. doi: 10.3389/fpsyg.2018.01471 (PMC6107842; doi:10.3389/fpsyg.2018.01471)

Figure 1. James and Mary interpersonal traits

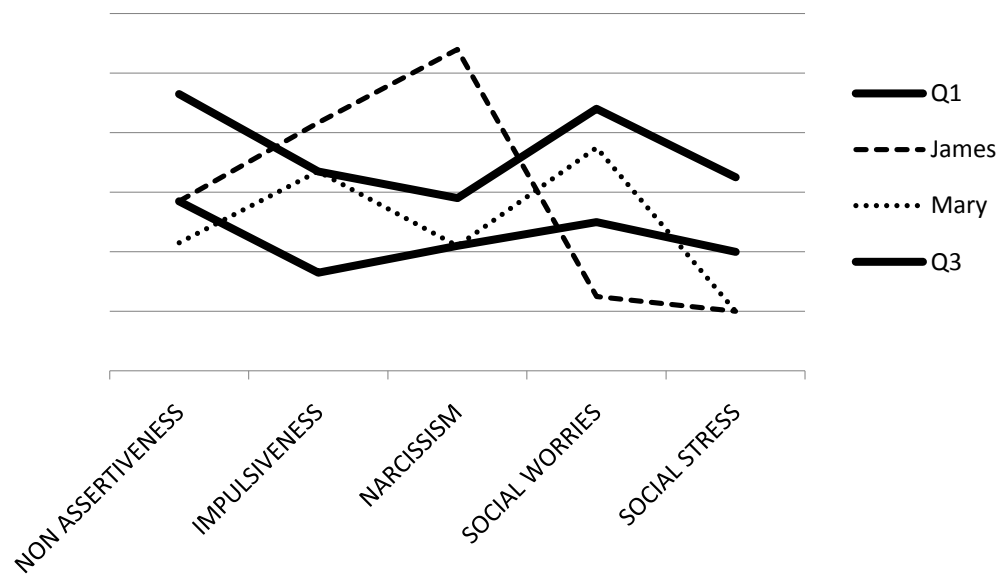

Supplement: Supplementary file 2 [file Image_1.PDF]
